# Healthcare of Indigenous Amazonian Peoples in response to COVID-19: marginality, discrimination and revaluation of ancestral knowledge in Ucayali, Peru

**DOI:** 10.1136/bmjgh-2020-004479

**Published:** 2021-01-07

**Authors:** Doreen Montag, Marco Barboza, Lizardo Cauper, Ivan Brehaut, Isaac Alva, Aoife Bennett, José Sanchez-Choy, Juan Pablo Sarmiento Barletti, Pilar Valenzuela, José Manuyama, Italo García Murayari, Miguel Guimaraes Vásquez, Celso Aguirre Panduro, Angela Giattino, Edwin Julio Palomino Cadenas, Rodrigo Lazo, Carlos A Delgado, Alfonso Nino, Elaine C. Flores, Maria Amalia Pesantes, Juan Pablo Murillo, Luisa Elvira Belaunde, Sergio Recuenco, Robert Chuquimbalqui, Carol Zavaleta-Cortijo

**Affiliations:** 1 Centre for Global Public Health, Queen Mary University of London, London, UK; 2 Centro de Investigaciones Tecnológicas, Biomédicas y Medioambientales - CITBM, Universidad Nacional Mayor de San Marcos, Lima, Peru; 3 Asociación Interétnica de Desarrollo de la Amazonia (AIDESEP), Lima, Peru; 4 ProPurus, Pucallpa, Peru; 5 Facultad de Salud Pública y Administración, Universidad Peruana Cayetano Heredia, Lima, Peru; 6 Vicepresidencia de Investigación, Universidad Nacional Intercultural de la Amazonia, Pucallpa, Peru; 7 Departamento Agroforestal Agrícola, Universidad Nacional Intercultural de la Amazonia, Pucallpa, Peru; 8 SHARE Amazónica, Pucallpa, Peru; 9 Department of World Languages and Cultures, Chapman University, Organge, California, USA; 10 Comité de Defensa del Agua, Iquitos, Loreto, Peru; 11 Red de Comunicadores Indígenas del Perú-Ucayali (REDCIP-U), Pucallpa, Peru; 12 Federación de Comunidades Nativas de Ucayali (FECONAU), Pucallpa, Peru; 13 Escuela de Sociologia, Facultad de Ciencias Sociales, Universidad Nacional Mayor de San Marcos, Lima, Peru; 14 Department of Anthropology, The London School of Economics and Political Science, London, UK; 15 Facultad de Ciencias del Ambiente, Universidad Nacional Santiago Antúnez de Mayolo, Huaraz, Peru; 16 Department of Anthropology, University of Massachusetts, Amherst, Massachusetts, USA; 17 Research Group Neonatology, Department of Pediatrics, Faculty of Medicine, Universidad Nacional Mayor de San Marcos, Lima, Peru; 18 Department of Medicine, Neonatal Unit, Instituto Nacional de Salud del Niño, Lima, Peru; 19 Facultad de Salud Pública, Universidad Peruana Cayetano Heredia, Lima, Peru; 20 Instituto de Investigación, Universidad Católica de Los Ángeles de Chimbote, Chimbote, Peru; 21 CRONICAS Center of Excellence in Chronic Disease, Universidad Peruana Cayetano Heredia, Lima, Peru; 22 Dickinson College, Carlisle, Pennsylvania, USA; 23 Departamento de Medicina Preventica y Salud Pública, Facultad de Medicina, Universidad Nacional Mayor de San Marcos, Lima, Peru; 24 Escuela Académico Profesional de Antropología, Universidad Nacional Mayor de San Marcos, Lima, Peru

**Keywords:** COVID-19, health systems

Systematic and persistent discrimination against Indigenous Peoples translates into differential health outcomes when analysed through ethnicity and/or mother tongue.^1^ In Peru, morbidity and mortality rates among Indigenous Peoples for COVID-19 appear to confirm this.^2^ The COVID-19 pandemic has highlighted the historical structural violence against Indigenous Peoples that currently takes a disproportionate toll in the Peruvian Amazon. This equally applies to Indigenous Andean Peoples and Afro Peruvians. Indigenous Peoples in voluntary isolation and those in initial contact are at highest health risk in this pandemic as they have no previous immunity against common infectious diseases, and lack access to public healthcare services. The Peruvian government introduced a state of emergency early on, but it did not work as theoretically expected because of the deeply rooted inequalities in Peru.

Public policies focused on reducing health inequities affecting Indigenous Peoples in peri-urban Amazonian contexts are urgently needed.^3^ Essentially, Indigenous Peoples (through their legitimate representatives) ought to be incorporated in the planning, monitoring, implementation and evaluation of those public policies to ensure sustainability, equity and inclusion in the short, medium and long run. It is also urgently necessary to rethink Peru’s health system to ensure it has an intercultural approach, designed for and with Indigenous Peoples in terms of prevention, treatment and access during and beyond the pandemic. An intercultural approach to healthcare implies that health services not only respects indigenous medical practices but promotes and enables joint and complementary interactions between biomedical and indigenous medical approaches to prevent and treat healthcare problems.^4^ In the last 15 years, Peru has produced more than 10 official documents on intercultural health, but very little of this has turned into practice. The problem is not the lack of an approach, as such, but the incapacity to turn it into practice.

Transforming the current health system to one where indigenous people have an active role and a direct governance mandate with specific budget allocation would be a step forward to address the current limitations in Peru’s health system in relation to providing equal access, prevention and treatment of Indigenous Peoples in urban, peri-urban and rural areas. For this, it is important to comprehend the context in which Indigenous Amazonian Peoples have experienced the pandemic, as we will outline below.

First, the pandemic occurs in an Amazonian context of biodiversity loss, rising deforestation, exposure to pollutants and forest fragmentation.^5^ This context increases the risk of future infectious disease emergencies and decreasing access to ancestral pharmacopoeia, central to Indigenous Peoples’ medical practices and a potential source of treatment for future re-emergence of infectious diseases across populations.^6^ Deforestation and forest fragmentation are driven by increased illegal timber, extractive economies and large-scale farming (especially African palm oil and coca leaf). These activities are cutting deeper into areas of the forest where there is no state presence or oversight, putting the lives and territorial sovereignty of Indigenous Peoples in risk, particularly those living in isolation, threatening their existence and right to isolation. Furthermore, deforestation and forest degradation leading to biodiversity loss threatens Indigenous Peoples’ food security, putting their health and well-being at an urgent risk.^7^ Food insecurity impacts microbiota homoeostasis with a direct impact on immune system development and response and therefore the ability to fight any infection.

Second, public policy must be developed from and with indigenous groups, including both female and male knowledge and initiatives. It should respect the biogeographic structure, with an intercultural, gender, intergenerational and multisectoral approach, taking into account not only the socioeconomic, sociocultural and environmental impact of the pandemic but also the risk resulting from socioeconomic isolation during the state of emergency which is challenging the livelihoods food security, individual health and child development of Amazonian families.

Third, given the current state of disinformation and alarming cases of self-medication across the Peruvian population,^8^ driven by inaccessible access to healthcare facilities, an increase of prices for basic fever medication, such as paracetamol (and other medications to treat COVID-19 symptoms), and an overwhelming number of infection rate and fatalities, there is a need of clear locally based healthcare responses. It is essential to train members of indigenous communities on clinical aspects and preventive measures related to the new pandemic.^9^ To strengthen the implementation of prophylaxis strategies and avoid the risks of taking medications without prior medical examination it is important to understand and acknowledge the cultural and linguistic heterogeneity of Indigenous Peoples, coproducing acceptable relief measures. There is also a need and an opportunity to use access and knowledge about local biodiversity and remedies to conduct research on a potential treatment, given that the majority of pharmaceuticals has a biological origin.

Faced with the difficulties of accessing health services, an indigenous health task force emerged: the *Matico* (Scientific name: *Buddleja globosa*. English: Matico pepper. https://www.minagri.gob.pe/portal/download/pdf/sectoragrario/agricola/lineasdecultivosemergentes/MATICO.pdf) command of Pucallpa. The *Matico* command is composed of volunteers from the Shipibo indigenous people, determined to use predominantly medicinal plants and therapies belonging to ancestral female healers, known as *raomi*, to provide personalised care to patients with mild symptoms who are unable to access health services. This grassroots initiative shows that local participation has empowered indigenous women to address health needs, especially when there is limited or no presence of health personnel in remote communities.^10^ Community participation in the context of COVID-19 also implies that prevention, care and management of deceased indigenous people will often be in people’s own hands and foster intergenerational dialogue as young people recover their grandmother’s healing knowledge. Here, the indigenous, and predominantly, female use of plants, has also enabled women to deal with the risks and consequences of an increase in domestic violence due to confinement conditions.

Local initiatives like the *Matico* have grown in other Amazonian cities, such as Sepáhua and Atalaya, always empowering women and older people’s knowledge to provide culturally relevant emotional and medical support over the last 6 months. It has also enabled coordination to ensure access to rapid tests, vital oxygen tanks and medicines, where regional government services failed coordinated and sufficient response. The *Matico* command appeared in a time when trust in the national healthcare system for the most marginalised people had been shattered mostly as a result of negative past experiences of discrimination and precarity of the healthcare system. The *Matico* command is a grassroots response vis-a-vis the incapacity of the national and regional governments and shows the importance of community engagement in developing the response. While this has been visible in urban areas, it becomes even more apparent in marginalised urban and in rural settings. There is an urgent need for rethinking the Peruvian healthcare system as a more participatory, community-engaged system that responds to local realities in a culturally appropriate way.

Fourth, this goes along with the increase of impact on mental health on local communities due to loss of family members, economic and food insecurity and the feeling of abandonment by the Peruvian government in times of emergency. Mental health services are already scarce and underfinanced in urban areas, even worse in rural areas. Intercultural mental health services addressing current needs within a pandemic context are urgently needed. Importantly, these need to be developed and addressed together with the community actors and should avoid a top-down approach and a purely biomedical approach.

Fifth, public planning policies need timely data to launch quick response actions, instead of reactive actions, as it is happening widely. Official epidemiological criteria were unable to follow since there was poor access to confirmation diagnostic test or contact trace capabilities in correlation with remoteness of some communities. It was impossible to perform and complete cases evaluation with the governmental criteria. A syndromic case definition and a community surveillance would have been a better approach for the indigenous context.^11^

Sixth, in the absence of a universal and inclusive health system,^12^ during the national quarantine ordered since 16 March, many indigenous people tried to return to the Ucayali region and their communities where they would have been able to and improve their survival strategies and have more access to indigenous preventive and therapeutic practices. Unlike those returning from abroad, internally displaced people received poor, inhuman and humiliating treatment, giving rise to new infections and perpetuating the historic marginalisation of Indigenous People.^13^

Finally, we are aware of the state efforts^14^ but also of the limitations of public health services and insufficient state support. For indigenous communities and intercultural urban settlements, marginalisation, poverty and racism continue. There is an urgent need for solidarity and support with indigenous movements to respond to the pandemic and think and work beyond the current pandemic in the prevention of future pandemics through biodiversity conservation and inclusive indigenous public policy and sovereignty. This ought to be done in a safe, ethical way, to prevent vulnerable people from being infected by people without verification of their COVID-19 status.^15^ Indigenous Peoples will continue demanding their rights, proposing concrete measures to confront COVID-19 and raising their voices at the national and international levels to ensure the protection of their people.
